# The ‘Dynamic’ Marriage Between Varicella and Zoster

**DOI:** 10.1016/j.ebiom.2015.09.014

**Published:** 2015-09-11

**Authors:** Pieter T. de Boer, Jan C. Wilschut, Maarten J. Postma

**Affiliations:** a Unit of PharmacoEpidemiology & PharmacoEconomics (PE2), Department of Pharmacy, University of Groningen, Groningen, Netherlands; b Institute of Science in Healthy Aging & healthcaRE (SHARE), University Medical Center Groningen (UMCG), Groningen, Netherlands; c Molecular Virology Section, Department of Medical Microbiology, University Medical Center Groningen (UMCG), Groningen, Netherlands; d Department of Epidemiology, University Medical Center Groningen (UMCG), Groningen, Netherlands

So-called dynamic modelling has explored various phenomena in infectious diseases' epidemiology and cost-effectiveness of interventions for control, and is increasingly becoming the standard and preferred approach in this area. As opposed to static modelling, dynamic modelling explicitly takes transmission of infectious agents into account, providing the opportunity to, for example, incorporate the indirect protective effect of vaccination on non-vaccinated individuals, i.e. herd protection, in the analysis (Jit and Brisson, 2011). Often, application of a dynamic model improves cost-effectiveness outcomes of infectious diseases control interventions, but in scarce cases detrimental effects on health, costs and cost-effectiveness might emerge. For example, vaccination may shift the age of infection upwards, which may be associated with increased severity of disease or associations with other diseases may exist (Heininger and Seward, 2006); reflecting additional effects that can be covered within a dynamic approach, next to herd protection. In this issue, Van Lier et al. (2015) add to such insights in the context of varicella vaccination, using dynamic modelling. In the context of modelling herd protection and age shifts, the authors illustrate the potentially crucial impact of varicella vaccination on herpes zoster (HZ) disease.

Varicella zoster virus (VZV) causes chickenpox during initial infection at childhood age. After resolution of the disease, however, the virus remains latently present in dorsal root ganglion cells. In later life, it can reactivate causing HZ. Potential relationship between varicella vaccination and HZ disease draws on the hypothesis raised by Hope-Simpson in the 1960s, that contacts between varicella-infected children and adults cause exogenous boosting of immunity against VZV in the latter, thus reducing the risk of HZ later in life (Hope-Simpson, 1965). In other words, childhood varicella vaccination, by eliminating exogenous boosting, might ultimately promote the risk of development of HZ in the adult population. This hypothesis is not undisputed, nor directly proven so far, but has received considerable attention. For instance, a recently published study from Ogunjimi et al. modelled that childhood varicella vaccination might almost double HZ incidence 30 years later (for example, among parents of vaccinated children) (Ogunjimi et al., 2015). Accordingly, it has been remarked – also by Van Lier et al. (2015) – that a few generations have to pay with an increased burden of HZ for the ultimate elimination of VZV on the long run.

In this issue, Van Lier et al. analysed the cost-effectiveness of varicella vaccination in the Netherlands using dynamic modelling with the Hope-Simpson hypothesis included and excluded (Van Lier et al., 2015). Considering a lifetime time horizon and discounting according to the Dutch guidelines for pharmacoeconomic research, the authors come to a clear and consistent conclusion. Without the Hope-Simpson hypothesis, varicella vaccination is highly cost-effective or even cost-saving with major gains of quality-adjusted life years (QALYs). Inclusion of the boosting hypothesis, however, results in dominance for the no varicella vaccination policy, because varicella vaccination would result in higher costs and QALY losses due to an increase of HZ in the adult population.

Prior to the current study of Van Lier et al., four studies have analysed the impact of varicella vaccination on HZ using dynamic modelling (Damm et al., 2015). These studies include investigations of the issue for surrounding countries of the Netherlands, such as the UK (van Hoek et al., 2012) and Belgium (Bilcke et al., 2013). Van Lier et al. support the overall findings from these earlier studies. Notably, similar to Van Lier et al., these studies generally found that varicella vaccination is not cost-effective or even results in net QALY losses when the Hope-Simpson hypothesis was included in the modelling. Generally, in these dynamic models cost-effectiveness of infant vaccination against varicella is driven by herd protection, related to the possible association with HZ and age shifts (upwards shift for varicella and potentially downwards for HZ). Potential improvement the health-economic profile of varicella vaccination, suggested by the authors, included targeting of vaccination to 11-year-old children after anamnesis (Damm et al., 2015).

Obviously, one solution to overcome the increase of HZ incidence among adults could be HZ vaccination (Damm et al., 2015, van Hoek et al., 2012, Bilcke et al., 2013), with highly effective vaccines being available and further developed. Van Lier et al. argue that HZ vaccination cannot be motivated from the perspective of its pushing varicella vaccination towards favourable cost-effectiveness. However, we would argue that HZ vaccination can be highly cost-effective on its own right and, on this basis, some countries, including the UK, have already implemented HZ vaccination. For the Netherlands, de Boer et al. (2013) estimated the cost-effectiveness of HZ-vaccination at €29,000–36,000 per QALY gained, depending on the vaccination age. At a cost-effectiveness threshold of €50,000 per QALY, these numbers may well be considered cost-effective, in particular in view of the fact that even higher thresholds of €80,000 per QALY or beyond apply to oncological and orphan drugs (Rozenbaum et al., 2010). Notably, the abovementioned studies for the UK (van Hoek et al., 2012) and Belgium (Bilcke et al., 2013) also found that combining paediatric varicella vaccination with HZ vaccination of the elderly would be cost-effective on the long run.

In conclusion, uncertainty on the cost-effectiveness of varicella vaccination remains, covering the full spectrum from being cost-saving to generating QALY losses. A change in this unsatisfactory situation through novel convincing epidemiological data cannot be expected on the short term. In the meanwhile, as suggested by Van Lier et al., decision-making on implementation of infant varicella vaccination in national immunization programmes should weigh the various scenarios within this ‘dynamic’ marriage between varicella and zoster, regarding costs, benefits and likelihoods of these scenarios. Notably, combination of childhood varicella vaccination with HZ vaccination of the adult population may reduce the uncertainty and represents a cost-effective option already per se.

## Conflict of Interest

MJP and JCW received grants and honoraria from various pharmaceutical companies, inclusive those developing, producing and marketing (HZ and varicella) vaccines. PTdB his position at the University of Groningen is financed by grants from Sanofi Pasteur, with the content of that work being unrelated to this editorial's topic.
